# Resident work hour restrictions do not improve patient safety in surgery: a critical appraisal based on 7 years of experience in Switzerland

**DOI:** 10.1186/1754-9493-6-17

**Published:** 2012-07-20

**Authors:** Adrian P Businger, Urban Laffer, Reto Kaderli

**Affiliations:** 1 Department of Surgery, Regionalspital Emmental AG, Oberburgstrasse 54, Burgdorf, CH-3400, Switzerland; 2 Emergency Department, University Hospital Bern, Bern, Switzerland; 3 Department of Surgery, Spitalzentrum Biel, Biel, AG, Switzerland

## Abstract

In 2005 the Swiss government implemented new work-hour limitations for all residency programs in Switzerland, including a 50-hour weekly limit. The reduction in the working hours of doctors in training implicate an increase in their rest time and suggest an amelioration of doctors' clinical performance and consequently in patients' outcomes and safety - which was not detectable in a preliminary study at a large referral center in Switzerland. It remains elusive why work-hour restrictions did not improve patient safety. We are well advised to thoroughly examine and eliminate the known adverse effects of reduced work-hours to improve our patients' safety.

## 

The two main issues of work-hour restrictions in medicine in Western countries were the improvement of patients’ safety and the doctors’ working conditions. Preceding the idea and implementation of restricted work hours and regulated work shifts were, exemplarily, the milestone Libby Zion case in 1984, some evidence that brings worsened results in clinical performance and simulated tasks [1,2], the view that overtired and inadequately supervised residents are a dangerous weak point in patient care [3], and the political will to approach the problem of doctors’ extensive work hours.

In consequence of this milestone case and based on grand jury recommendations, the New York State Department of Health implemented junior doctors’ work hours limitations restricted to 80 hours a week in 1989 and subsequently, the US Accreditation council for Graduate Medical Education implemented nationwide duty-hours standards by July 1, 2003 with some modifications in 2008 [4-6].

In the United Kingdom restrictions on residents’ work hours were introduced in 1996 with a progressive reduction in junior doctors’ working hours based on the European Working Time Directive [7]. On January 1, 2005 the Swiss government implemented new work-hour limitations for all residency programs in Switzerland [8]**,** including a 50-hour weekly limit with a maximum overtime of 2 hours per day and 140 hours per year, respectively, and at least 11 hours of rest between duty periods. Overtime per day may exceed 2 hours during work-free business days or in emergency cases. Daily rest time may be reduced to 9 hours several times a week, as long as the resting time amounts to 12 hours, averaged over 2 weeks.

The reduction in the working hours of doctors in training implicate an increase in their rest time and suggest an amelioration of doctors’ clinical performance and consequently in patients’ outcomes and safety. This seems very plausible and is furthermore supported by some evidence in standardized tasks in the lab. However, in real-world conditions the anticipated positive effect of reduced working hours on patients’ care is not detectable. Therefore, several questions arise: whether the reduction of working hours has been adequately implemented by the departments, how strict the residents’ adherence to the restrictions is, and whether residents will use their off-time effectively for recovering; it is well known that work hours are frequently underreported by residents [9]. Two reviews provide compelling evidence that residents are more rested after implementation of work-hour restrictions [10,11]. It remains unclear whether the studies are able to show what they intend: Even when methodological weaknesses (frequently single training sites and non-randomized studies) of the included studies might impair the conclusions in two recent systematic reviews, a reduction in working hours to 80 hours has neither shown a clear positive effect on patients’ outcomes and safety, nor a clear negative effect of working in shifts, with self-evidently more handovers, transfers and a possible less continuity of patient care; and the systematic reviews have shown only a limited effect on postgraduate medical education [12,13]. Ultimately, it cannot be distinguished whether this neutral effect is due to a non-detrimental effect of work hour restriction or is counterbalanced by improved patient care and resident supervision. It remains elusive why work-hour restrictions did not improve patient safety. It seems that there are other influencing factors, such as loss of flexibility or monetary aspects, above all in the United States [14]. The same amount of work should be performed in restricted time while there is insufficient financial support.

The above-mentioned conclusions are all drawn from studies originating in the United States, with a reduction in working hours to 80 hours per week; data from mainland Europe, with reduced hours to less than 56 or 48 hours a week in the United Kingdom, are scarce and contradictory [15-17]. If we assume again that fewer working hours produce more rest time and less fatigue, a more sufficient restriction in work hours from arbitrary 80 hours to 50 hours a week might produce a greater improvement in patients’ outcome and safety. In a preliminary study at a large referral center in Switzerland, using the prospective database of the Association for Quality Assurance in Surgery (AQC), the patients‘ morbidity and mortality following common surgeries, operated by residents before (2001–2004; 1259 patients) and after (2005–2008; 1427 patients) the effects of implementation of work-hour limitations were assessed. The in-hospital mortality (Odds Ratio [OR] 3.61, 95% Confidence Interval [CI] 1.01-12.93, p < 0.05) and postoperative surgical complication rate (OR 2.76, 95% CI 1.61-4.73, p < 0.01) were significantly higher after the reform. No significant differences could be found concerning the overall intraoperative (p = 0.61) and postoperative medical complication frequencies (p = 0.08) [18]. This study has several shortcomings, such as retrospective analysis, single-center site study, non-randomized, and a relatively small “n”. Furthermore, the working hours for doctors-in-training decreased from 52.5 (standard deviation [SD] 0.7) before to 49.7 (SD 0.7) hours after the reform. However, an improvement in patient safety has not occurred with the more stringent Swiss work-hour regulation. Work-hour limitations for residents are taken for granted. Studies assessing the effects of work-hour restrictions originating from mainland Europe with greater reductions in the number of work hours compared to those in the United States are essential.

## Conclusions

Considering the different number of work hours for doctors-in-training in the United States and in Switzerland and the failure of improvement in patients’ outcome and safety with 80 hours as well as 50 hours, the focus of regulatory agencies and labor unions should change from hours worked to other aspects within work-hour limits (e.g unpaid extracontractual work). Reflecting the critics of decreased professionalism due to less responsibility it might be pathbreaking to qualitatively assess arguments among decision-makers what the main impediments to reduction in working hours are. We are well advised to thoroughly examine and eliminate the known adverse effects of reduced work-hours to improve our patients’ safety.

## Competing interests

UL and RK are employed by the Spitalzentrum Biel. UL is the Director of Surgery at Spitalzentrum Biel. The authors declare no other competing interests related to this editorial.

## Authors’ contributions

All authors contributed equally to drafting this editorial, and read and approved the final version of the manuscript.
